# Building a dynamic correlation network for fat-tailed financial asset returns

**DOI:** 10.1007/s41109-016-0008-x

**Published:** 2016-08-02

**Authors:** Takashi Isogai

**Affiliations:** Bank of Japan, 2-1-1 Nihonbashi-Hongokucho, Chuo-ku, Tokyo, 103-8660 Japan

**Keywords:** Asset returns, Dynamic correlation, Fat-tail, Volatility model, Filtering, Correlation network

## Abstract

In this paper, a novel approach to building a dynamic correlation network of highly volatile financial asset returns is presented. Our method avoids the spurious correlation problem when estimating the dynamic correlation matrix of financial asset returns by using a filtering approach. A multivariate volatility model, DCC–GARCH, is employed to filter the fat-tailed returns. The method is proven to be more reliable for detecting dynamic changes in the correlation matrix compared with the widely used method of calculating time-dependent correlation matrices over a fixed size moving window, which can have fundamental problems when applied to fat-tailed returns. We apply the method to selected Japanese stock returns to observe the dynamic network changes as a case study. The estimated time-dependent correlation matrices are then compared with those calculated by using the traditional method to highlight the advantages of the proposed method. Two types of indicators, namely the largest eigenvalue and cosine distance measures, are introduced to identify significant changes in the correlation matrix for an initial screening of remarkable stress events. A more detailed network-based analysis is then conducted by examining topological measures calculated from the network adjacency matrices. The higher density and lower heterogeneity of the correlation network during stress periods are clearly observed, while the correlation network of stock returns is shown to be robust with regard to time. The method discussed in this paper is not limited to stock returns; it can also be applied to build a dynamic correlation network of other financial and non-financial time series with high volatility.

## Introduction

A correlation network is a network whose adjacency matrix is built on the basis of pairwise correlations between variables. In the context of financial asset returns including stock prices and exchange rates, contemporaneous co-movement plays a key role in portfolio optimization and risk management. We, therefore, focus on an undirected and weighted correlation network in this study. The concept of a dynamic network is that the adjacency matrix changes dynamically: the network structure can change depending on time. In financial markets, the correlation between asset prices can change dynamically in response to the trading activities of market participants, occasionally driven by unexpected environmental changes. Those changes can be described as changes in the structure of the correlation network. Because monitoring and analyzing such dynamic changes can help improve investment technology and risk management as well as our understanding of the market structure, we are interested in the dynamic correlation network in the context of financial asset returns.

When building a dynamic correlation network of asset returns, we need to set up a time series of the correlation matrices. The most important challenge is creating a correlation matrix, which is then converted into a weighted adjacency matrix by applying numerical operations including weight conversion and thresholding. Specifically, the choice of correlation measure and calculation method of the conditional correlation is crucial. If the estimated correlation matrix is distorted, any analysis based on the correlation network can be misleading.

To measure the co-movement of financial asset returns, Pearson’s linear correlation measure is frequently used. The correlation matrix of asset returns is built on Pearson’s linear correlation in many quantitative financial models; however, we should be careful to calculate the linear correlation, since it can cause a serious distortion problem when used for fat-tailed financial time series including stock returns and exchange rates, which have significant volatility changes. Indeed, the spurious correlation caused by volatility shock is a typical problem that leads to a false signal about the co-movement.

Another important point to consider when calculating the time series of sample correlation matrices is how to choose an observation period. The true correlation between two asset returns at a specific time is not directly observable; therefore, we need to estimate it from the observable sample data. The most widely used practical method is to calculate the sample correlation by employing a moving window method, in which the correlation is calculated statically with the data over a fixed observation period (e.g., a moving window of 30 trading days). The window may be overlapped or non-overlapped, depending on the need for a smaller or larger number of correlations. The correlation matrix should be updated continuously by rolling the window; however, the moving window method makes the correlation measure less responsive to the current price co-movement. Past large shocks can still have a significant impact on the correlation until they are excluded from the window.

The combination of these two methods, namely the correlation matrix of unfiltered sample data series with a moving window, thus has fundamental problems that are widely acknowledged by researchers and practitioners; however, the method is still widely used because of the lack of an alternative.

In this regard, we propose a novel approach to building a dynamic correlation network for highly volatile financial asset returns. We focus on estimating the correlation matrix since this process is the dominant part of building a correlation network. The main contribution of this paper is to provide an efficient and reliable way in which to build a series of correlation matrices for the analysis of dynamic networks (e.g., the inter-temporal comparison of networks). This approach can be widely applied for preprocessing highly volatile time series data in the context of dynamic network analysis. The method employs the dynamic correlation of asset returns that is estimated by using a multivariate volatility model, namely the generalized autoregressive conditional heteroskedasticity model with dynamic conditional correlation (DCC–GARCH). Since the correlation matrix has a dynamic structure in the model, the distortion caused by large volatility fluctuations is greatly reduced. We also propose a way in which to monitor dynamic changes in the correlation matrix through eigenvalue analysis: the intensity and direction of the correlation are separated and examined independently to detect any significant change. Further, we apply the proposed method to selected Japanese stock returns to build dynamic correlation networks in order to analyze their dynamic topological changes.

## Literature review

The earlier study by Mantegna (1999) developed a correlation network of US stock returns, which provided the basis for a network-based approach toward financial data analysis. They first calculated the cross correlation of returns in terms of Pearson’s linear correlation and then constructed a minimum spanning tree (MST) to discover the hierarchical structure of the network. Similar network-based analyses have since been conducted by many researchers including Onnela et al. (2003a), Onnela et al. (2003b), and Chi K et al. (2010).

Some of these studies have focused on the dynamic aspects of the correlation network to assess how the network changes. Onnela et al. (2003a) focused on the dynamics of market correlations. They built a time-dependent MST for stock return data on the New York Stock Exchange and concluded that the basic topological structure of the network is robust with respect to time, while strong market correlation is identified during crisis periods. Bonanno et al. (2004) investigated various stock portfolios at different time horizons to observe the correlation structure by examining how returns are affected by the time horizons used to compute the correlation. Kenett et al. (2010) analyzed stationary correlations between stocks by using a visualization tool, namely the Stock Market Holography, that allows an investigation of the structure and dynamics of the market through the study of correlations.

These previous research works have used Pearson’s linear correlation of the sample returns, which is also used to calculate the dynamic correlation by shifting the observation period. In this study, however, we focus more on the method of calculating the asset return correlation, since the synchronized volatility shocks of fat-tailed asset returns can affect the level of Pearson’s linear correlation significantly. Specifically, these volatility shocks can distort the correlation structure when a market-wide shock occurs. In our previous research (Isogai 2014), we applied a multivariate volatility model to control for volatility fluctuations in order to avoid such a distortion problem when clustering a static correlation network. In this study, we use a more advanced type of volatility model with dynamically changing correlation (DCC–GARCH) to control for such volatility fluctuations. The conditional correlation matrices are estimated by using the DCC–GARCH model, which enables us to create a dynamic correlation network. We also propose measures to track the correlation dynamics to identify any significant change in the asset return correlation.

The remainder of this paper is organized as follows. Section “Asset correlation and volatility model” describes the technical problems regarding the correlation of fat-tailed returns and proposes the use of the multivariate volatility model. Section “Estimation of the dynamic correlation” describes the result of the model fitting to Japanese stock market data. Section “Eigenvalue analysis of the dynamic correlation” describes the framework of the dynamic analysis of the correlation matrix by using eigendecomposition. Section “Discussion” discusses the advantages of the proposed approach as well as some difficulties from a dynamic network analysis perspective. Section “Conclusion” concludes.

## Asset correlation and volatility model

### Linear correlation and volatility fluctuations

Pearson’s linear correlation is frequently used to indicate the degree of the co-movement of two asset returns. Pearson’s linear correlation *ρ*_*X*,*Y*_ is defined as

1 $$ \rho_{X,Y}=\frac{\text{Cov}\left(X,Y\right)}{\sqrt{\text{Var}(X)\cdot\text{Var}(Y)}}   $$

where Var(·) and Cov(·) are the variance and covariance operators, respectively.

There is another type of linear correlation, namely the partial correlation that captures the direct influence between two variables, thereby eliminating the indirect influence via other variables. The partial correlation is useful for measuring the direct and indirect linkages between asset returns. Some previous research works including Kenett et al. (2010) and Kenett et al. (2015) have used the partial correlation to explore the underlying structures of stock markets. In this research, we focus on the standard correlation, since the overall interaction including indirect ones should be considered for many financial operations including portfolio optimization and risk quantification. The method proposed below, however, can be applied for the use of the partial correlation by converting the estimated standard correlation into the partial one.

**Fig. 1:**
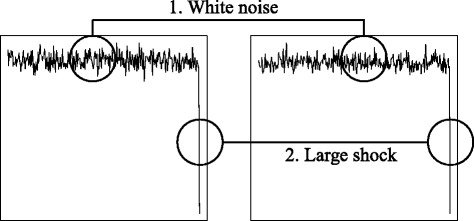
Synchronized Volatility Shock of Asset Returns. Note: A pair of uncorrelated white noise series with length 100 is generated by random sampling from the bivariate standard normal distribution. A pair of noise (-10, -10) is added as simultaneous large shocks. The sample correlation increases from 0.00 to 0.57 in this case

The use of Pearson’s linear correlation implicitly assumes that the two variables are normally distributed. The distribution of financial returns, however, frequently shows fat-tailed features with many extreme values. Moreover, volatility changes dynamically and large fluctuations in returns tend to cluster together, resulting in the persistence of high volatilities (i.e., volatility clustering). These features of asset returns can significantly distort Pearson’s linear correlation. Figure 1 illustrates a simple example of the distortion problem. The linear correlation rises significantly when an extremely large value is added to the two uncorrelated random noise series. The distortion effect of a volatility shock on linear correlation may cause the spurious correlation problem as shown by this example.

A rank correlation measure of returns, including Spearman’s *ρ* and Kendall’s *τ*, can be alternatives. These rank correlations are non-parametric measures that are dependent only on the rank order of variables; therefore, they do not assume a linear function between two variables as in Pearson’s linear correlation. They can also be transformed into Pearson’s linear correlation under certain conditions. The problem, however, is that the rank order of the variables is not always preserved when the volatility factor is removed from the returns (i.e., rank inconsistency).

Other types of correlation measures include mutual information, which relates to the joint entropy of two variables. The probability density functions of returns and residuals are not necessarily the same; therefore, this approach can have a similar problem to the rank correlation.

Hence, volatility fluctuations should be controlled for when estimating a correlation matrix. In this regard, modeling volatility fluctuations explicitly is more reliable than applying a non-linear correlation measure directly to fat-tailed returns when estimating a correlation matrix that is converted into an adjacency matrix.

Further, the linear correlation has important practical advantages when used properly. For example, many mathematical models for pricing and risk modeling as well as portfolio optimization are built with linear correlations in multivariate settings. Such models can become more complicated if the non-linear type of correlation is built-in.

### Model-based dynamic correlation

A time series of correlation matrices is frequently calculated by using the moving window method. The correlation matrices can be used as an input for creating dynamic adjacency matrices; however, the method has serious drawbacks when capturing the dynamic features of correlation changes. The correlation is calculated to indicate the degree of co-movements of two variables within an observation window; therefore, past large shocks can have significant impacts on the correlation until the event goes out of the window. Once the correlation has increased considerably because of a large shock, a higher level of correlation is maintained even if the shock has already disappeared. This is the distortion effect of volatility shocks along the time axis, which can generate serious noise when observing the dynamic changes in the correlation. This distortion effect can be mitigated by adopting a shorter observation window; however, this approach may hamper the stability of the sample correlation. Positive-definiteness is another important condition that restricts the length of the observation period (i.e., matrix rank condition).

These problems can be solved by employing a model-based dynamic correlation approach. Specifically, we choose the DCC–GARCH model proposed by Engle (2002), which can separate the volatility fluctuations from returns, leaving the standardized residuals, which are independently and identically distributed (i.i.d.). The model thus implements the dynamics of the correlation matrix change, which enables us to introduce a model-based dynamic correlation of returns without using the moving window method. Such a dynamic correlation matrix is expected to be more responsive; therefore, the dynamic correlation network analysis of asset returns would become more precise and convincing.

In general multivariate GARCH models, a vector of asset returns ***r***_*t*_ is decomposed into the conditional mean and volatility as

2 $$ \begin{aligned} &\boldsymbol{r}_{t}=\boldsymbol{\mu}_{t}+\boldsymbol{\varepsilon}_{t}=\boldsymbol{\mu}_{t}+\boldsymbol{H}_{t}^{1/2}\boldsymbol{z}_{t},\\ &\mathrm{E}\left(\boldsymbol{z_{t}}\right)=\boldsymbol{0},\ \text{Var}\left(\boldsymbol{z_{t}}\right)=\boldsymbol{I}_{N}\\ \end{aligned}   $$

where ***μ***_*t*_ is a vector of the conditional means, ***ε***_*t*_ is a vector of the unpredictable residuals, ***H***_*t*_ is an *N*×*N* (*N*: the number of returns) symmetric positive-definite matrix, which is a conditional variance–covariance matrix of ***r***_*t*_, and ***z***_*t*_ is a vector of i.i.d. standardized residuals, the mean and variance of which are ***0*** and ***I***_*N*_ (an identity matrix of order *N*), respectively.

DCC–GARCH has three parts: the mean part, volatility part, and DCC part. The mean part is modeled by using the autoregressive moving average (ARMA) model independently as

3 $$ \boldsymbol{\mu}_{t} =\boldsymbol{\mu} +\sum\limits_{i=1}^{P} \boldsymbol{A}_{i} \boldsymbol{r}_{t-i} +\sum\limits_{j=1}^{Q} \boldsymbol{B}_{j} \boldsymbol{\varepsilon}_{t-j}   $$

where ***A***_*i*_ and ***B***_*j*_ are diagonal matrices. The volatility part is modeled as

4 $$ \boldsymbol{h}_{t} =\boldsymbol{\omega} +\sum_{i=1}^{q}\boldsymbol{S}_{i}\boldsymbol{\varepsilon}_{t-i} \odot \boldsymbol{\varepsilon}_{t-i} +\sum_{j=1}^{p}\boldsymbol{T}_{j}\boldsymbol{h}_{t-j}   $$

where ⊙ denotes the Hadamard operator (the entry-wise product), ***h***_*t*_ is the diagonalized matrix of ***H***_*t*_, and both ***S***_*i*_ and ***T***_*j*_ are diagonal matrices. Volatility is modeled without interaction between the assets to simplify the model.

In the DCC part, an *N*×*N* positive-definite dynamic correlation ***R***_*t*_ is used to model the dependency structure of ***r***_*t*_; the more formal definition of ***R***_*t*_ is described later in model fitting. The time-dependent structure of ***R***_*t*_ is described by using a proxy variable, which is introduced to ensure the positive-definiteness of ***R***_*t*_. The proxy variable ***Q***_*t*_ is modeled as

5 $$ \begin{aligned} \boldsymbol{Q}_{t} &=\boldsymbol{\bar{Q}}+\sum_{i=1}^{m}a_{i}\left(\boldsymbol{z}_{t-i}\boldsymbol{z}_{t-i}^{'}-\boldsymbol{\bar{Q}}\right)+\sum_{j=1}^{n}b_{j}\left(\boldsymbol{Q}_{t-i}-\boldsymbol{\bar{Q}}\right)\\ \end{aligned}   $$

where *a*_*i*_ and *b*_*j*_ are non-negative scalars and $\boldsymbol {\bar {Q}}_{t}$ is the unconditional mean of ***Q***_*t*_. The DCC model with time lags in the conditional correlation is denoted as DCC (*m*, *n*). The parameter *a*_*i*_ shows the sensitivity of ***Q***_*t*_ to previous shocks, while the parameter *b*_*j*_ represents the persistence of the correlation in previous periods. The correlation matrix ***R***_*t*_ is calculated by rescaling ***Q***_*t*_ as

6 $$ \boldsymbol{R}_{t}=\text{diag}\left(\boldsymbol{Q}_{t}\right)^{-\frac{1}{2}}\boldsymbol{Q}_{t}\text{diag}\left(\boldsymbol{Q}_{t}\right)^{-\frac{1}{2}}.   $$

The positive-definiteness of ***Q***_*t*_ and ***R***_*t*_ is ensured by the following conditions:

7 $$ a_{i} \ge 0,\quad b_{j} \ge 0, \quad \sum_{i=1}^{m}a_{i}+\sum_{j=1}^{n}b_{j}<1.   $$

Thus, the vector of asset returns ***r***_*t*_ in (2) is modeled by DCC–GARCH with (3), (4), and (6). For more details on the DCC–GARCH model, see Engle and Sheppard (2001) and Engle (2002).

Volatility fluctuations are separated from returns ***r***_*t*_, reducing the risk of the spurious correlation problem. The dynamic correlation matrix ***R***_*t*_ shows the point-in-time pairwise correlation, which is expected to be more responsive than that calculated directly from the sample return ***r***_*t*_ by using the moving window method. Further, ***R***_*t*_ is ensured to have positive-definiteness at all times.

## Estimation of the dynamic correlation

### Stock return data

We use two sample datasets of Japanese stock returns for the empirical study. The 50 largest companies listed on the First Section of the Tokyo Stock Exchange are selected from two industries, namely transportation equipment (manufacturing) and banking (services), based on their market capitalization ranking, respectively. The sample portfolio of transportation equipment includes many automobile companies as well as parts and peripherals companies; the sample portfolio of banks includes large internationally operating banks as well as smaller regional banks. The data frequency is daily; the period runs from the beginning of January 2008 to the end of June 2015, which includes the two major financial shocks of the recent past: the Lehman collapse (2008) and the Great Earthquake (2011). Price data are converted into log returns.

### Model fitting

To estimate the model parameters of DCC–GARCH by maximum likelihood estimation, the likelihood function needs to be identified. The distribution of ***z***_*t*_ in (2) has not been specified. We assume one of the normal, Student *t*, and skew *t* distributions, allowing for some fat-tailedness even after the volatility adjustment. Such a heterogeneous distributional assumption makes it difficult to use multivariate distribution as the joint distribution function of ***r***_*t*_ in the likelihood function; thus, we employ a copula-based approach. The copula function *C*(·) is defined as

8 $$ F\left(x_{1},\,\ldots,\, x_{N}\right)=C\left(F_{1}\left(x_{1}\right),\,\ldots,\, F_{N}\left(x_{N}\right)\right)  $$

where *F*(·) is the joint distribution function of the variables ***X***=(*X*_1_,…,*X*_*N*_) (Sklar’s theorem Sklar (1959)). The joint density function *f*(*x*) of ***X*** can be described as

9 $$ \begin{aligned} f\left(x_{1},\,\ldots,\, x_{N}\right) &=c\left(F_{1}\left(x_{1}\right),\,\ldots,\, F_{N}\left(x_{N}\right)\right)\prod_{i=1}^{N}f_{i}\left(x_{i}\right) \end{aligned}   $$

where *f*_*i*_(*x*_*i*_) is the marginal distribution of *x*_*i*_ and *c*(·) is the density function of the copula. Specifically, we choose the Student *t*-copula that can handle tail dependency. Thus, the dependence structure of the margins is assumed to follow a Student *t*-copula with conditional correlation ***R***_*t*_ and constant shape parameter. For technical details on the Student *t*-copula, see Demarta and McNeil (2005).

Hence, the joint density function of ***r***_*t*_ is defined as a combination of the copula density and density of the i.i.d. residual ***z***_*t*_ as

10 $$ \begin{aligned} &f\left(\boldsymbol{r}_{t}|\boldsymbol{\mu}_{t},\ \boldsymbol{h}_{t},\ \boldsymbol{R}_{t},\ \eta\right)\\ &=c^{S_{t}}\left(u_{1{\cdot}t},\ \ldots,\,\ u_{N{\cdot}t}|\boldsymbol{R}_{t},\ \eta\right) \prod_{i=1}^{N}\frac{1}{\sqrt{h_{i{\cdot}t}}}f_{i{\cdot}t}\left(z_{i{\cdot}t}|\theta_{i}\right) \end{aligned}   $$

where *u*_*i*·*t*_=*F*_*i*_(*r*_*i*·*t*_|*μ*_*i*·*t*_,*h*_*i*·*t*_,*θ*_*i*_), *θ*_*i*_ is a parameter set including the ARMA–GARCH parameters in (3) and (4) and distributional parameters of *z*_*i*_, *c* ^*S*^_*t*_(·) is the Student *t*-copula density function, and *η* is the shape parameter of the Student *t*-copula. In the DCC setting, we assume the time-dependent structure of ***R***_*t*_ as described in (5) and (6). The estimate of ***R***_*t*_, therefore, collapses to non-negative scalars *a*_*i*_ and *b*_*j*_ as defined in (5).

The log-likelihood function *L* *L*(***θ***|***r***_*t*_) is built by using (10), which can be separated into the copula part with the DCC parameters (***a***, ***b***) and marginal distribution part. The two parts of the log-likelihood function can be maximized independently: first, the individual distributional parameter set *θ*_*i*_ is estimated, followed by the DCC parameters (***a***, ***b***). The model selection is based on the AIC. This two-stage estimation procedure is especially convenient when the number of assets becomes large. For more technical details about the two-stage maximum likelihood estimation, see Patton (2006) and Joe (2005).

### Estimation results of the DCC

Table 1 shows the estimation results for the DCC parameters of the two sector-based sample portfolios. The DCC–GARCH model is fitted independently to each portfolio in order to investigate the correlation dynamics in these sectors. Note that these sample portfolios are created representing typical manufacturing and service sectors, which may have different correlation dynamics with different DCC parameters. It is also possible to create much larger sample portfolios that cover multiple sectors, although model fitting can be harder.

**Table 1:** DCC estimation result

Sector	m, n	a1	b1	b2	b1+b2	*η*
Transportation	1, 2	0.0061	0.4161	0.4493	0.8654	30.4160
equipment		(0.0007)	(0.0808)	(0.0805)		(1.2898)
Banking	1, 2	0.0094	0.3940	0.4461	0.8402	21.5952
		(0.0009)	(0.0686)	(0.0695)		(1.0033)

Note: The DCC order (*m*, *n*) and parameters *a*_1_, *b*_1_, and *b*_2_ are defined in (5). *η* is the shape parameter of the Student *t*-copula in (10). The R (http://cran.r-project.org/) package “rmgarch” (Ghalanos 2014) is used for the parameter estimation

The selected DCC order is (1, 2) for both portfolios. The choice of DCC order depends on the AIC as mentioned above. The larger value of *b*_1_+*b*_2_ means that the dynamic correlation matrix ***R***_*t*_ is more dependent on its past values than previous shocks, since the parameter *b*_*j*_ represents the degree of persistence of the correlation. The positive-definiteness condition shown in (7) is confirmed to be satisfied. For the ARMA–GARCH model of individual returns defined in (3) and (4), the distribution of *z*_*i*_ is mostly Student *t*. Other details of the estimation results are omitted because of space limitations.

## Eigenvalue analysis of the dynamic correlation

Two vectors of the dynamic correlation matrix ***R***_*t*_ are successfully estimated by fitting the DCC–GARCH model to the stock returns of the two sectors. In addition, we also calculate two vectors of dynamic correlation matrix $\boldsymbol {R}^{m}_{t}$ from the sample returns by Pearson’s linear correlation using the moving window method with a 200 trading day window. We then conduct an inter-temporal analysis of the dynamic correlation of stock returns by using ***R***_*t*_ and $\boldsymbol {R}^{m}_{t}$.

### Intensity and direction of the correlation matrix

It is difficult to observe the time series trend of ***R***_*t*_ as it is in matrix form. We hence need the dimension reduction of ***R***_*t*_. In this regard, the eigendecomposition of a matrix is useful; the matrix can then be represented in terms of its eigenvalues and eigenvectors:

11 $$ \boldsymbol{R}_{t}=\boldsymbol{U}_{t}\boldsymbol{\varLambda}_{t}\boldsymbol{U}_{t}^{-1}   $$

where ***U***_*t*_ is the *N*×*N* matrix whose *i*-th column is the eigenvector ***u***_*i*·*t*_ of ***R***_*t*_, while ***Λ***_*t*_ is the diagonal matrix whose diagonal elements are the corresponding eigenvalues as *λ*_*i*·*t*_=***Λ***_*ii*·*t*_. We assume that *λ*_*i*·*t*_ and ***u***_*i*·*t*_ are sorted in descending order: *λ*_1·*t*_ is the largest eigenvalue and ***u***_1·*t*_ is the eigenvector that corresponds to *λ*_1·*t*_.

Geometrically, an eigenvector points in the direction of the corresponding eigenvalue; the eigenvalue represents the scaling factor alongside the direction. In the context of the correlation matrix, the eigenvalue can be regarded as the correlation intensity of the correlation matrix, while the eigenvector shows the multidimensional direction. It is, therefore, possible to summarize the information contained in the correlation matrix ***R***_*t*_ by using ***Λ***_*t*_ as an intensity measure and ***U***_*t*_ as a direction measure. Only the largest eigenvalue *λ*_1·*t*_ and associated eigenvector ***q***_1·*t*_ might be sufficiently meaningful when the group comprises homogeneous variables. Otherwise, in the case of networks with heterogeneous subgroups or communities, the remaining ones may be sufficiently relevant to play important roles in the networks. The point here is on which eigenvalues and associated eigenvectors to focus in order to monitor the inter-temporal changes in ***R***_*t*_.

In this regard, random matrix theory can help determine which eigenvalue and eigenvector should be selected for monitoring. The limiting distribution of the largest eigenvalue of a randomized return correlation matrix with the same size as ***R***_*t*_ is obtained as the Tracy–Widom distribution (Johnstone 2001); therefore, some high percentile value of the distribution can be used as the threshold above which the eigenvalue of a correlation matrix is meaningful. For the level of percentile value, we adopt the 99th percentile. Another well-known distribution, the Mar$\breve {c}$enko–Pastur distribution, suggests the boundary of the distribution of eigenvalues. The 99th percentile of the Tracy–Widom distribution is larger than that of the Mar$\breve {c}$enko–Pastur distribution. For more mathematical details on eigenvalues and the Tracy–Widom and Mar$\breve {c}$enko–Pastur distributions, see Johnstone (2001) and Tracy and Widom (1994, 1996, 2009).

### Cosine distance measure of the eigenvectors

While the eigenvalue is a scalar value that can be directly compared at different times, we need some measure to compare eigenvectors. In this regard, we use a cosine distance measure that defines the distance between two numerical vectors. First, the cosine similarity between vector ***x*** and ***y*** is defined as

12 $$ cos(\theta)=\frac{\boldsymbol{x}\cdot \boldsymbol{y}}{\left\Vert \boldsymbol{x}\right\Vert \left\Vert \boldsymbol{y}\right\Vert }=\frac{\sum x_{i}y_{i}}{\sqrt{\sum {x_{i}^{2}}\sum {y_{i}^{2}}}}   $$

Then, the cosine distance measure is defined as

13 $$ \gamma(\boldsymbol{x},\ \boldsymbol{y}) = 1-cos(\theta)   $$

As the benchmark of the comparison, we use the eigenvector $\boldsymbol {\bar {u}}_{i}$ of the unconditional correlation matrix $\bar {\boldsymbol {R}}$ calculated over the whole observation period. The cosine distance is denoted as $\gamma (\boldsymbol {u}_{i\cdot t},\ \boldsymbol {\bar {u}}_{i})$. Similarly, the eigenvector $\boldsymbol {\bar {u}}^{m}_{i}$ of $\bar {\boldsymbol {R}}^{m}$ is used as the benchmark to measure the cosine distance $\gamma ^{m}(\boldsymbol {u}^{m}_{i\cdot t},\ \boldsymbol {\bar {u}}^{m}_{i})$ for the moving window method. The cosine distance is expected to capture significant changes in the direction of the eigenvector compared with that of the benchmark eigenvector.

The cosine distance is normalized by its standard deviation, since it takes a very small number,

14 $$ \nu_{i\cdot t} = \frac{\gamma(\boldsymbol{u}_{i\cdot t},\ \boldsymbol{\bar{u}}_{i})}{\text{std}(\gamma_{i})}   $$

where *ν*_*i*·*t*_ is the rescaled cosine distance between the *i*-th eigenvector *q*_*i*·*t*_ of ***R***_*t*_ and the *i*-th eigenvector $\boldsymbol {\bar {u}}_{i\cdot t}$ of $\bar {\boldsymbol {R}}_{t}$, while std(*γ*_*i*_) is the sample standard deviation of $\gamma (\boldsymbol {u}_{i\cdot t},\ \boldsymbol {\bar {u}}_{i})$ over the observation period. *ν*_*i*·*t*_ is calculated at every point in time *t* during the observation period. $\nu ^{m}_{i\cdot t}$ is defined similarly for the moving window method.

The normalized cosine distance *ν*_*i*·*t*_, especially *ν*_1·*t*_, is useful to detect significant changes in the structure of a correlation matrix. In the context of network analysis, any relative change in link weights between nodes can be detected by the cosine distance *ν*_1·*t*_, while a systematic increase in the correlation over the whole network is captured by the correlation intensity *λ*_1·*t*_. Such a structural change in the correlation network can be local or global.

### Comparison between the dynamic correlation and moving window

The important variables for the comparison between the model-based dynamic correlation and moving window are as follows: *λ*_1·*t*_ as the largest eigenvalue of the dynamic correlation ***R***_*t*_; $\lambda ^{m}_{1\cdot t}$ as the largest eigenvalue of the correlation matrix $\boldsymbol {R}^{m}_{t}$; *ν*_1·*t*_ as the cosine distance of the eigenvector *u*_1·*t*_ of ***R***_*t*_; and $\nu ^{m}_{1\cdot t}$ as the cosine distance of the eigenvector $u^{m}_{1\cdot t}$ of $\boldsymbol {R}^{m}_{t}$.

Table 2 shows the result of the eigendecomposition of ***R***_*t*_ and $\boldsymbol {R}^{m}_{t}$ of the transportation equipment and banking sectors. The range of eigenvalues during the observation period for the largest and the second largest, *λ*_*i*=1,2·*t*_ and $\lambda ^{m}_{i=1,2\cdot t}$, are shown. The 99th percentile values of the Tracy–Widom distribution as well as the Mar$\breve {c}$enko–Pastur are also calculated.

**Table 2:** Eigenvalues of the dynamic correlation matrix

		Eigenvalue			99th percentile	
		Largest	Second largest		TW	MP
		(min - max)	(min - max)			
Transportation equipment						
	Dynamic (*λ*_1·*t*_,*λ*_2·*t*_)	24.28 - 27.17	0.97 - 1.40		1.38	1.33
	Moving Average ($\lambda ^{m}_{1\cdot t}, \lambda ^{m}_{2\cdot t}$)	19.97 - 31.39	1.32 - 2.67			
Banking						
	Dynamic (*λ*_1·*t*_,*λ*_2·*t*_)	31.56 - 35.20	1.25 - 1.73		1.38	1.33
	Moving Average ($\lambda ^{m}_{1\cdot t}, \lambda ^{m}_{2\cdot t}$)	25.89 - 39.60	0.84 - 2.70			

Note: The eigenvalues of ***R***_*t*_ are calculated on every trading day during the observation period. Min and max represent the minimum and maximum of the vector of the corresponding eigenvalues, respectively. TW represents the Tracy–Widom distribution; MP represents the Mar$\breve {c}$enko–Pastur distribution

The range of the largest eigenvalues, *λ*_1·*t*_ and $\lambda ^{m}_{1\cdot t}$, during the observation period is far above the 99th percentile of the Tracy–Widom distribution in either sector. For the second largest eigenvalues, *λ*_2·*t*_ and $\lambda ^{m}_{2\cdot t}$, the maximum value is higher than the 99th percentile point, whereas the minimum value is lower than that point. These eigenvalues appear to have only a limited impact compared with the dominant impact of the largest ones; however, some community structure may exist in these two sectors. A hierarchical group structure exists in many correlation networks of financial returns ((Mantegna 1999; Tumminello et al. 2010), and (Mitrović and Tadić 2009)). Here, we follow only the largest eigenvalues and associated eigenvectors, considering the clear difference in the eigenvalues between the largest and the second largest.

Another finding is that the range of eigenvalues $\lambda ^{m}_{i=1,2\cdot t}$ is wider than *λ*_*i*=1,2·*t*_. This finding means that $\boldsymbol {R}^{m}_{t}$ tends to take larger eigenvalues and have greater fluctuation than ***R***_*t*_, probably because of the volatility fluctuations of returns as mentioned earlier. Thus, $\boldsymbol {R}^{m}_{t}$ can have a higher risk of spurious correlation problem.

Table 3 shows the range of the cosine distance *ν*_1·*t*_ and $\nu ^{m}_{1\cdot t}$. *ν*_1·*t*_ takes a much wider range compared with $\nu ^{m}_{1\cdot t}$. The dynamic correlation is unaffected by volatility fluctuations; therefore, a large value of *ν*_1·*t*_ can provide reliable hints to detect the timing of a correlation change more efficiently than $\nu ^{m}_{1\cdot t}$. This is another advantage of the model-based dynamic correlation matrix.

**Table 3:** Cosine distance of the largest eigenvector

	Cosine distance
	(min - max)
Transportation equipment	
Dynamic (*ν*_1·*t*_)	0.00 - 12.99
Moving Average ($\nu ^{m}_{1\cdot t}$)	0.47 - 5.15
Banking	
Dynamic (*ν*_1·*t*_)	0.00 - 21.66
Moving Average ($\nu ^{m}_{1\cdot t}$)	0.34 - 4.90

Note: Cosine distance *ν* is defined in (14)

### Changes in the largest eigenvalue of the dynamic correlation

**Fig. 2:**
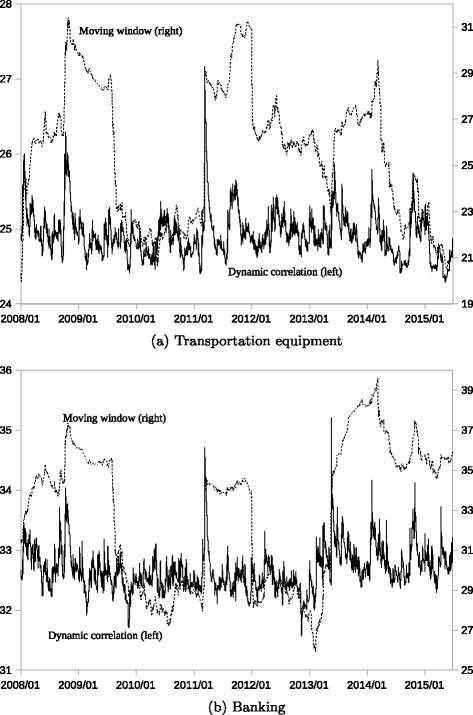
Changes in the Largest Eigenvalues of the Dynamic Correlation. Note: The *solid line* represents the largest eigenvalue *λ*_1·*t*_ of the dynamic correlation matrix ***R***_*t*_ estimated by the DCC–GARCH model in the left scale; the *dotted line* represents the largest eigenvalue $\lambda ^{m}_{1\cdot t}$ of the moving window-based correlation matrix $\boldsymbol {R}^{m}_{t}$ in the right scale

Figure 2 shows the time series trend of the largest eigenvalues, *λ*_1·*t*_ and $\lambda ^{m}_{1\cdot t}$, which represent the correlation intensities of the correlation matrices, ***R***_*t*_ and $\boldsymbol {R}^{m}_{t}$, respectively.

An important observation is that $\lambda ^{m}_{1\cdot t}$ (the dotted line) stays at a higher level once a large increase is observed. As expected, *λ*_1·*t*_ (the solid line) appears to be responsive, while $\lambda ^{m}_{1\cdot t}$ is significantly affected by its past increases. For example, significant increases in *λ*_1·*t*_ are observed in 2008 (the Lehman collapse) and 2011 (the Great Earthquake) for both sectors. In both cases, the solid line falls after the events, while the dotted line tends to lag behind the movement of the solid line. Such a large increase has a persistent impact on the future level of $\lambda ^{m}_{1\cdot t}$ as long as it is included in the observation period. In this case, the window size is 200 trading days. The lagging effect accumulates when multiple increase events occur; then, the correlation intensity appears to increase significantly, whereas the actual intensity may have already decreased, as indicated by *λ*_1·*t*_.

The dynamic correlation matrix ***R***_*t*_ tells us that a sharp increase in the correlation intensity is observed after the market disturbances; however, such an increase tends to be contained quickly. Conversely, $\boldsymbol {R}^{m}_{t}$ indicates that a higher correlation intensity is persistent, longer than the actual. Thus, the pattern of changes in the correlation intensity differs between ***R***_*t*_ and $\boldsymbol {R}^{m}_{t}$.

If we build a network adjacency matrix from $\boldsymbol {R}^{m}_{t}$, the degree and timing of changes in the correlation intensity will not be correctly identified. Hence, the analysis of dynamic networks can be significantly distorted. This limitation is a major drawback to using the moving window-type calculation method of the correlation matrix.

### Changes in the cosine distance measure of the dynamic correlation

**Fig. 3:**
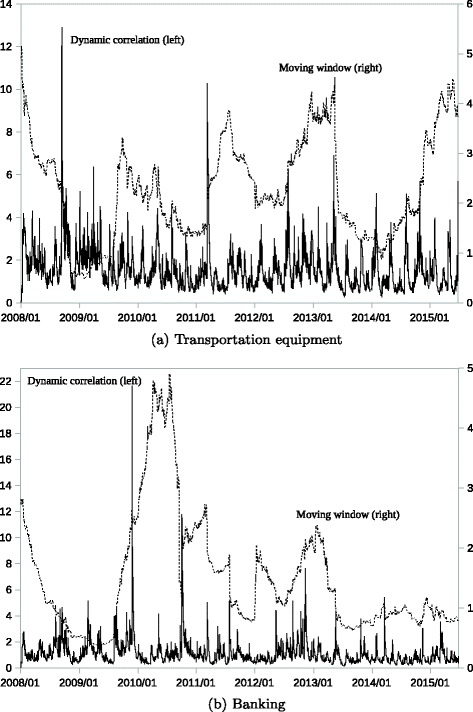
Changes in the Cosine Distance of the Dynamic Correlation. Note: The *solid line* represents the cosine distance *ν*_1·*t*_ of the dynamic correlation ***R***_*t*_ in the *left scale*; the *dotted line* represents the cosine distance $\nu ^{m}_{1\cdot t}$ of the moving window correlation matrix $\boldsymbol {R}^{m}_{t}$ in the *right scale*

Figure 3 shows the time series trend of the cosine distance, *ν*_1·*t*_ (the solid line) and $\nu ^{m}_{1\cdot t}$ (the dotted line), which indicate changes in the direction of the correlation matrices ***R***_*t*_ and $\boldsymbol {R}^{m}_{t}$, respectively. Note that changes in the cosine distance correspond to changes in the relationship between individual asset returns.

*ν*_1·*t*_ appears to distinguish the possible timing of a correlation change. Change point detection is easier to pick up the sharp hikes of the solid line. On the contrary, $\nu ^{m}_{1\cdot t}$ has a similar problem to the correlation intensity in that it seems to be affected by past events. The dotted line suggests the same timing of a change as the solid line indicates in some cases; however, it is difficult to identify the exact start and end points just from the dotted line.

In Fig. 3 a (transportation equipment), the solid line clearly shows the sharp increases after the Lehman collapse (2008) and the Great Earthquake (2011). This means that not only the sharp increases in intensity shown by Fig. 2 a but also the significant changes in direction occurred at that time in the transportation equipment sector. This sector has two subgroups: large automobile companies and medium-sized parts companies. In the crisis periods, the stock prices of the former group responded more than those of the latter group. This difference between the two groups caused temporal correlation changes in direction.

In Fig. 3 b (banking), the solid line clearly shows the sharp increases in 2009 and 2010. In Fig. 2 b, the sharp increases in the correlation intensity are observed after the Lehman collapse and the Great Earthquake, which means systematic changes that affect the entire banking sector. On the contrary, *ν*_1·*t*_ responded little after those crisis events. In 2009 and 2010, significant changes in monetary policy were introduced to enhance monetary easing in Japan. The stock prices of some regional banks responded more to these monetary policy changes, since such regional banks have more financial assets that are affected by the policy change than large internationally operating banks. The differences in stock price responses thus caused a temporal change in the cosine distance in the banking sector.

The changes in *ν*_1·*t*_ allow us to detect those temporal changes, although we need more detailed analysis to explain the background of what occurred. When we conduct network-based analysis using the estimated correlation matrix, the information available from the cosine distance as well as the correlation intensity helps narrow the observation period efficiently to enhance the detail of the analysis. The key point is to use the dynamic correlation ***R***_*t*_, since the moving window method has serious drawbacks that may cause the misleading selection of the target period.

## Analysis of dynamic network changes

The largest eigenvalue and normalized cosine distance calculated from the corresponding eigenvector proved to be reliable measures to trace the dynamic changes of the correlation matrix. We thus build a dynamic correlation network from the estimated correlation matrices to study the possible topological changes in the network. We use a simple unsigned nondecreasing adjacency conversion based on the estimated correlation matrix ***R***_*t*_:

15 $$ \boldsymbol{A}_{ii,t}=0, \quad \boldsymbol{A}_{ij\left(i\neq j\right),t}= \left\{ \begin{array}{ll} |cor\left(x_{i},\, x_{j}\right)_{t}|\ &\quad if\ \,\, cor\left(x_{i},\, x_{j}\right)_{t}>0\\ 0 &\quad if\ \,\, cor\left(x_{i},\, x_{j}\right)_{t}\leqq0\\ \end{array}\right.   $$

where ***A***_*ij*,*t*_ is the (*i*, *j*)_*th*_ entry of the conditional weighted adjacency matrix ***A***_*t*_; *c* *o* *r*(*x*_*i*_, *x*_*j*_)_*t*_ is the (*i*, *j*)_*th*_ entry of the dynamic correlation matrix ***R***_*t*_. Note that an edge has connection weight in a weighted network, while an edge takes only 1 or 0 value (connected or not) in an unweighted network. The diagonal elements of ***A***_*ij*,*t*_ is 0, since any self-edge is not considered in the correlation network.

An undirected weighted network only with edges positively weighted is built by (15). We are mainly interested in the co-movement of stock prices; therefore, negative correlation information is not retained. Moreover, no thresholding is implemented for a positive correlation to keep as much information as possible for calculating the topological measures; we also aim to avoid technical difficulties to set the threshold level, which is beyond the scope of our study.

Handling a negative correlation when it exists can be difficult. We assume that it is sufficient to focus on the positive correlation of returns; however, a negative correlation may have some meaning when discussing the diversification of investment. Hence, the way in which we handle negative correlations depends on the context of the analysis and algorithm to be applied. For stock return data, a negative correlation is far less frequently observed than a positive correlation. Indeed, no negative pairwise correlation exists in either sector in our dataset during the observation period; thus, the weighted adjacency matrix is basically the same as the correlation matrix with regard to the non-diagonal part. Further, there are many other types of adjacency conversions including the power function such as ***A***_*ij*,*t*_=|*c* *o* *r*(*x*_*i*_, *x*_*j*_)_*t*_|^*β*^. We have not tested any other conversion functions; this is an important caveat to our analysis.

For every conditional network adjacency matrix, we calculate the following three topological measures. First, network density *D*(***A***) is defined as the mean of the off-diagonal elements of weighted adjacency matrix ***A***:

16 $$ D(\boldsymbol{A}) = \frac{\sum_{i}\sum_{j>i}\boldsymbol{A}_{ij}}{n\left(n-1\right)/2}=\frac{mean\left(\boldsymbol{k}\right)}{n-1}\approx\frac{mean\left(\boldsymbol{k}\right)}{n}   $$

where ***k*** is a vector of the node degree (connectivity), *m* *e* *a* *n*(·) is the arithmetic mean function, and *n* is the number of nodes. The node degree *k*_*i*_ is defined as the sum of the row or column of an adjacency matrix:

17 $$ k_{i}=\sum_{j\neq i}\boldsymbol{A}_{ij}  $$

The connectivity *k*_*i*_ equals the sum of connection weights between node *i* and the other nodes. Network density *D*(***A***) measures the overall connection (correlation) among nodes. A density close to 1 indicates that all nodes are strongly correlated with each other. We also calculate network centralization *C*(***A***) and heterogeneity *H*(***A***), which are defined as below:

18 $$ \begin{aligned} C(\boldsymbol{A})&=\frac{n}{n-2}\left(\frac{max\left(\boldsymbol{k}\right)}{n-1}-\frac{mean\left(\boldsymbol{k}\right)}{n-1}\right)\\ &=\frac{n}{n-2}\left(\frac{max\left(\boldsymbol{k}\right)}{n-1}-D(\boldsymbol{A})\right)\approx\frac{max\left(\boldsymbol{k}\right)}{n}-D(\boldsymbol{A})\\ \end{aligned}   $$

19 $$ H(\boldsymbol{A})=\frac{\sqrt{var\left(\boldsymbol{k}\right)}}{mean\left(\boldsymbol{k}\right)}=\sqrt{\frac{n\sum_{i}{k_{i}^{2}}}{\left(\sum_{i}k_{i}\right)^{2}}-1}   $$

Centralization *C*(***A***) is 1 when one node has fully connected edges with all other nodes that are not connected with each other. It is 0 for a network where each node has the same connectivity. Network heterogeneity *H*(***A***) measures the variation in connectivity across nodes. It is defined as the coefficient of variation of the connectivity distribution.

These three topological measures are calculated for the conditional adjacent matrix ***A***_*ij*,*t*_ for every trading day during the observation period. For more details on these topological measures, see Horvath (2011).

### Dynamic changes in network topological properties

Table 4 summarizes the network properties of the two sectors. The density measures the overall relationship among individual stocks in a correlation network. The density of the banking network is higher than that of the transportation equipment network. It means that the nodes of the banking sector network are more tightly connected with each other than those of the transportation equipment sector, while almost every node is connected (correlated) with the rest of nodes in both correlation networks.

**Table 4:** Network properties of the correlation networks by sector

		Transportation equipment				Banking		
		Density	Centralization	Heterogeneity		Density	Centralization	Heterogeneity
		*D*(***A***)	*C*(***A***)	*H*(***A***)		*D*(***A***)	*C*(***A***)	*H*(***A***)
Minimum		0.468	0.102	0.110		0.618	0.059	0.078
Mean		0.481	0.117	0.126		0.641	0.069	0.088
Maximum		0.528	0.121	0.134		0.694	0.076	0.101
S.d.		0.006	0.002	0.003		0.007	0.002	0.002

Note: Density *D*(***A***), centralization *C*(***A***), and heterogeneity *H*(***A***) are calculated by (16), (18), and (19), respectively. The minimum, maximum, and mean are calculated from the time series of the three measures. S.d. denotes standard deviation

The levels of centralization are very low in both sectors. When centralization is close to 0, it means that many of the nodes in a network are almost equally connected; no one particular node is located at the center of the network. The low values of heterogeneity in both sectors mean that these networks seem to be highly homogeneous, since heterogeneity indicates the variation in connectivity across nodes. Banking is less centralized than transportation equipment, while transportation equipment is more heterogeneous than banking.

**Fig. 4:**
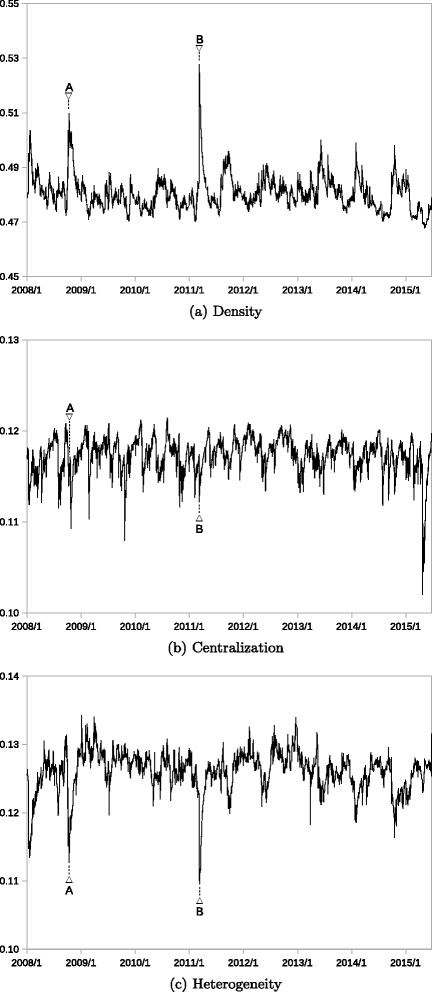
Network Properties (Transportation Equipment). Note: Density, centralization, and heterogeneity are calculated by (16), (18), and (19), respectively. A and B correspond to the stress events listed in Table 5

**Fig. 5:**
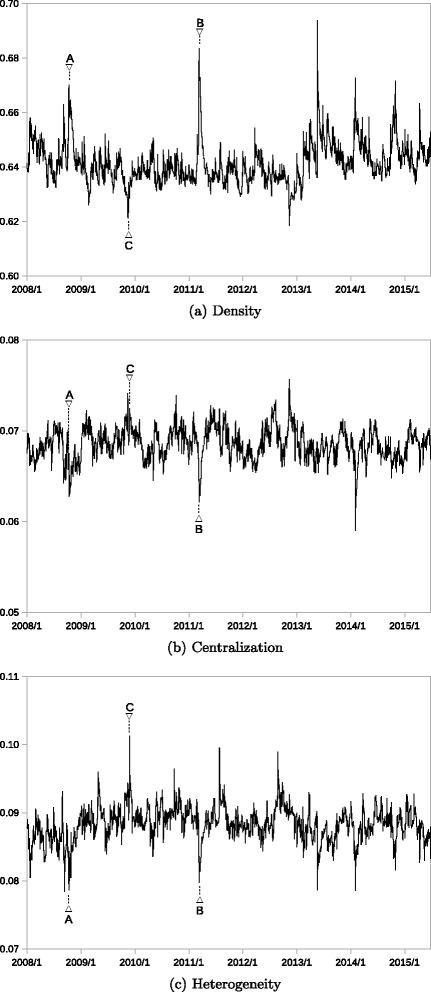
Network Properties (Banking). Note: Density, centralization, and heterogeneity are calculated by (16), (18), and (19), respectively. A, B, and C corresponds to the stress events listed in Table 5

Figures 4 and 5 depict how these three topological measures of the two networks changed during the observation period. Two or three stress events were selected based on the timing when the largest eigenvalue or cosine distance recorded large values, as shown in Figs. 2 and 3. The trading days of these stress events are listed in Table 5. The network topological properties are analyzed in detail at these selected timings.

**Table 5:** Stress events for the dynamic comparative analysis

Trading date	Event	Transportation	Banking
		equipment	
15/10/2008	Lehman collapse	A	A
1/12/2009	Monetary policy change	-	C
15/3/2011	Great Earthquake	B	B

Note: The Lehman collapse and Great Earthquake are selected as global (market-wide) stress events that increased the volatility of many stocks significantly; the monetary policy change event is selected as the banking sector-specific ones identified by the clear hike in the cosine distance shown in Fig. 2 b. The marks A, B, and C are identifiers of events referred to in the figures hereafter.

Figure 4 depicts the development of the topological measures of the transportation equipment network. Figure 4 a illustrates that the density of the network increased significantly during the two stress events, namely the Lehman collapse (marked by A) and the Great Earthquake (marked by B), while it remained at similar levels in the other periods. The increase is larger when the Great Earthquake occurred than during the Lehman collapse. These changes in network density are consistent with the increases in the largest eigenvalues shown by Fig. 2 a. Note that the volatility factor is properly controlled for by GARCH modeling; therefore, such higher densities in the correlation network reveal that the connectivity between nodes intensifies when a stress event occurs. In the context of the factor contribution to stock prices, individual (idiosyncratic) factors may be less influential, whereas the systematic factor contributes more.

On the contrary, centralization exhibits a limited range of fluctuation during the observation period; it remained at around the same level throughout the period, as shown in Fig. 4 b. Even when the two stress events occurred, no significant change in centralization was observed. This low level of centralization means that the network has no centralized structure, suggesting that every node in the network is affected significantly by unobservable common factors.

Heterogeneity sharply dropped during the two stress events, as shown in Fig. 4 c. When these stress events occurred, the variance in the correlation decreased because of a breakdown in the temporal correlation; the mean of the correlation increased as indicated by the higher density of the network. Such changes contributed to lower heterogeneity, as defined in (19). Intuitively, the network becomes more homogeneous with smaller local differences in the network structure.

Figure 5 depicts the development of the topological measures of the banking network. Figure 5 a shows that the density increased significantly when the two stress events (marked by A and B) occurred, similar to in the transportation equipment network. Another significant increase is identified when the monetary policy change occurred in 2009 (marked by C), which is particular to the banking sector. These increases are also consistent with the changes in the largest eigenvalues shown in Fig. 2 b.

Centralization remained within a limited range, as shown by Fig. 5 b, again as in the transportation equipment network. Centralization, however, is much lower than that in the transportation equipment network, suggesting that the nodes are more equally connected. The banking network comprises a small number of large banks and many smaller regional banks; the lower centralization of the network may thus reflect the tight connection between regional banks. Centralization dropped during the Great Earthquake, while no significant change was observed when the Lehman collapse occurred.

Figure 5 c shows that heterogeneity dropped significantly during the two stress events, as observed in the transportation equipment network. Further, heterogeneity increased sharply in 2009 (marked by C), as is clearly identified by the cosine distance measure shown in Fig. 3 b. Similar hikes in heterogeneity were observed in 2011 and 2012. These hikes in heterogeneity are related to market events that caused local correlation increases in the banking sector; no similar events were observed in the transportation equipment sector at that time.

In summary, the common observation of the changes in network properties is that density increased significantly when the stress events occurred together with the lower level of heterogeneity. Changes in centralization seem to be less evident compared with the other two topological measures. Moreover, the two networks remained stable in non-stressed periods, although some occasional changes in network properties did occur.

The changes in the largest eigenvalues are consistent with the changes in network density in both sectors. The cosine distance measure works fine to detect changes in the network properties such as centralization and heterogeneity during stress periods. It also detects local changes in the local network structure, suggesting that some irregular event is occurring. The analysis shows that the combined usage of the two eigenvector-based measures can track dynamic network changes in a compact way.

### Comparison between static and dynamic correlation networks

**Fig. 6:**
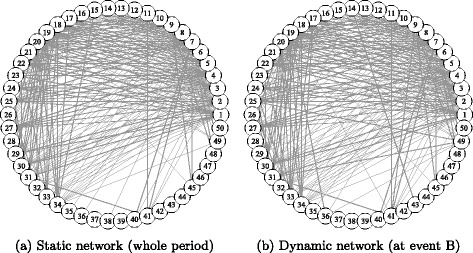
Transportation Equipment Network. Note: The nodes are sorted by descending order of market capitalization at the end of the observation period: node 1 has the largest market capitalization. The node positions of the two networks are the same. Only the edges with a weight larger than the 75th percentile of the edge weight distribution are displayed. Event B of the dynamic network is defined in Table 5

**Fig. 7:**
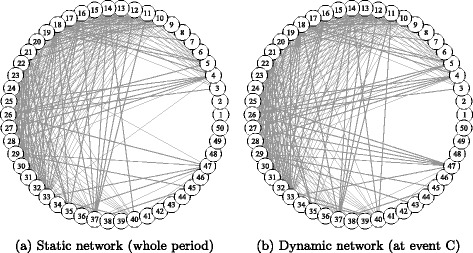
Banking Network. Note: The nodes are sorted by descending order of market capitalization at the end of the observation period: node 1 has the largest market capitalization. The node positions of the two networks are the same. Only the edges with a weight larger than the 75th percentile of the edge weight distribution are displayed. Event C of the dynamic network is defined in Table 5

In the previous section, the dynamic topological changes of the two networks were analyzed. In this section, we compare the static network representation (as a benchmark network) based on the whole observation period with the snapshot of the dynamic network at some stress event (at time *s*) to examine if there are any significant differences. Figures 6 and 7 show the static and dynamic networks, representing the static adjacency matrix $\boldsymbol {\bar {A}}_{ij}$ and the conditional one ***A***_*ij*|*t*=*s*_, respectively. For stress event selection, the timing when the cosine distance significantly increased allows us to detect any global or local change in the network. We select just one stress event for each sector as a typical example for the comparative analysis.

The correlation network is similar to a complete graph with almost every node connected; therefore, only the upper 25 percent of all edges with a higher level of edge weight are displayed to observe the highly connected part of the network. The nodes are located in the same positions in the static and dynamic networks. The node IDs are sorted in descending order of market capitalization (analogous to corporate size).

Interestingly, the static networks of transportation equipment and banking appear to differ greatly. This means that the network structures of the two sectors are fundamentally different, at least when evaluated by using the corporate size factor. The network of transportation equipment appears to be densely connected with the nodes ranked higher in terms of capitalization (smaller node IDs), as shown in Fig. 6 a. Many are global car manufacturers, which reside in the center position of the network and are more densely connected with each other.

On the contrary, the banking network is densely connected around the nodes with middle ranks of capitalization. Specifically, the largest three nodes (globally operating banks) are not connected with other medium and lower ranked nodes. The network shown in Fig. 7 a basically comprises regional banks, which seem to form a tightly connected subnetwork. This observation is consistent with the findings of our previous research (Isogai 2014).

This comparison of the static and dynamic networks in each sector reveals some differences between these two types of networks, even though they appear to be largely similar. This finding means that the network structures are almost stable in both sectors, but that considerable changes are observed occasionally.

In transportation equipment, the network appears to be densely connected with the group of the largest nodes as mentioned above. Figure 6 b shows the snapshot of the dynamic network captured at the Great Earthquake (event B). The difference between the two networks is that the edges are more widely distributed between nodes in the dynamic network. Further, some lower ranked nodes have more edges in the dynamic network; such wider distribution of edges is consistent with its decreased heterogeneity, as shown in Fig. 4 c.

In banking, the snapshot of the dynamic network is captured at event C, when the largest cosine distance value was observed in Fig. 3 b. Large globally operating banks show little correlation with smaller regional banks. Thus, the network is densely connected among medium ranked nodes, while sparsely connected with higher and lower ranked nodes. In the dynamic network, some nodes have more edges, especially in lower ranked nodes (smaller capitalization) than in the static network; moreover, heterogeneity and centralization increased significantly when the event occurred, as shown in Fig. 5 b and c. This finding means that connections between specific nodes are intensified temporarily by market events, as mentioned earlier.

The above two examples show that a large cosine distance can detect different types of changes in network structure. Heterogeneity and centralization may respond differently to the large increase in the cosine distance; therefore, a more detailed analysis of network properties is required once the timing is identified by initial screening with the cosine distance. Further, the detection of temporal and local changes is difficult by only comparing the static and dynamic network representation, since the change is not necessarily clearly revealed. The cosine distance measure is thus helpful for selecting those periods in which to find any subtle differences.

## Discussion

First, the dynamic correlation method can provide an efficient framework for creating a time series of the correlation matrices. The presented case study based on Japanese stock price data reveals that the model-based dynamic correlation matrix is useful for detecting and analyzing inter-temporal changes in a correlation matrix efficiently. The dynamic correlation estimation by using the DCC–GARCH model has many advantages compared with the widely used moving window-based calculation method. Specifically, the more responsive feature of the DCC-based dynamic correlation is useful and reliable. Further, it overcomes the problem of the moving window-based correlation that the effect of past large shocks persists longer and distorts the current correlation. The moving window-based method has a higher risk of spurious correlations because of the distortion effect by volatility fluctuations, which is properly controlled for under the DCC–GARCH model. Further, the DCC–GARCH model has a rigorous theoretical background, in which the positive-definiteness condition is always ensured. On the contrary, the DCC-based dynamic correlation also has some shortcomings including its complicated estimation process. For example, we tested a small portfolio of just 50 stocks; however, the model fitting process would become challenging if the number of assets increased further still.

When building a dynamic correlation network of asset returns, the most important aspect is estimating the correlation matrix, since all the information about the network is stored as an adjacency matrix derived from the correlation matrix. We also need to create a time series of adjacency matrices to follow the evolution of the asset correlation network. The data size, therefore, would be much larger than the static one. What is important here is the data dimensionality reduction, enabling a compact expression of the network without losing any important information about network changes for a detailed comparative analysis of the network. If we can identify the exact timing of an important change to be focused on automatically (change point detection), it is possible to compress the adjacency matrix data including only the interested observation periods. Hence, efficient data compression alongside the time axis becomes available. We can then apply more complicated methods to the reduced network data to retrieve more knowledge from the same dataset.

Another point is the separation of the correlation intensity and correlation distance to analyze changes in a correlation matrix. When we are interested in any correlation change, a change in intensity or direction has a different meaning from the network perspective. A change in intensity has a systematic impact on the entire network; all nodes are affected unanimously, whereas the linkages between them are basically the same except for the link weight values. When we are interested in the changes in the whole network, this type of change has an important meaning. A change in direction indicates that something new or irregular has occurred in the network structure. The change covers just one part of the network or the entire network. Looking at the changes at an individual data point level may provide more details about them. It is therefore crucial to follow the two types of different changes (i.e., intensity and direction) to carry out an efficient dynamic network analysis. Our eigendecomposition approach for the separation of intensity and direction is proven to work well by the case studies in this regard. Nevertheless, a more advanced topic regarding the eigendecomposition, namely the stability of the decomposition, needs to be examined from the viewpoint of the inherent noise problems in correlation matrices, as discussed in Tumminello et al. (2010).

The two dynamic networks converted from the correlation matrices are analyzed by examining the changes in network topologies. The higher densities and lower heterogeneity of the network are commonly observed during crisis periods, while centralization is less responsive compared with these two measures. The combination of higher density and lower heterogeneity indicates the fragile structure of a network, which can lead to a larger risk of stock portfolios. The analysis of topological measures ensures that the network became riskier when stress events occurred.

While such changes in the network structure are outstanding, they did not last long in any stress period; the network seems to be stable and retain a similar structure over the longer term. This means that the correlation between the stocks in the two sectors is robust with respect to time, as shown by previous research (Onnela et al. 2003a). The network may switch between the two regimes of crisis and non-crisis situations. It is thus meaningful to extend the analysis to the other sectors to confirm if the network structures remain stable. A more detailed analysis with other topological measures would also be meaningful and informative.

It is confirmed that the largest eigenvalue and cosine distance summarize the topological measures of density, centralization, and heterogeneity well. Network density is almost parallel to the largest eigenvalues. Moreover, the cosine distance measure summarizes the local and global structural changes in the network. The cosine distance measure thus appears to be useful for the initial screening of the dynamic changes of the network topology. We can delve into more details once we have identified the timing of any possible changes in the network. Indeed, monitoring burden would be greatly reduced when applied to risk monitoring in stock markets.

Many other types of correlation networks including MST can be built from the same estimated correlation matrix by applying more complicated adjacency conversion procedures. Other types of correlation measures including the partial correlation can also be used to build a correlation network. Further analysis is thus needed to confirm if the same result is obtained under different types of network building approaches.

## Conclusion

In this study, we propose a novel approach to building a dynamic correlation network of fat-tailed asset returns by using the DCC–GARCH model, which can control for volatility fluctuations to avoid the spurious correlation problem. A model-based dynamic correlation matrix is estimated by fitting the model to Japanese stock returns data on two industries, which are then compared with another correlation matrix calculated by the traditional moving window method. The case study shows that the dynamic correlation method describes the dynamic changes in a more responsive way, while the correlation calculated by the moving window method has serious distortion problems. Moreover, temporal changes in the correlation network with higher density and lower heterogeneity are clearly observed; the result is convincing and consistent with the findings of earlier research works.

In future research, we will analyze the dynamic correlation network of Japanese stock returns in more detail. Specifically, a wider coverage of inter-temporal comparative analysis of network structure is of interest. Community detection in specific industries and shock propagation analysis during crisis periods are also important topics to be covered. Further, the change point detection and data compression technique proposed in this paper will be examined more precisely. Finally, the research framework of the dynamic correlation estimation and analysis can be applied to other financial and non-financial networks, which have high levels of volatility fluctuations.
